# Insights Into Ferroptosis, a Novel Target for the Therapy of Cancer

**DOI:** 10.3389/fonc.2022.812534

**Published:** 2022-02-25

**Authors:** Hong-Tao Wang, Jie Ju, Shao-Cong Wang, Yu-Hui Zhang, Cui-Yun Liu, Tao Wang, Xue Yu, Fei Wang, Xue-Ru Cheng, Kun Wang, Zhao-Yang Chen

**Affiliations:** ^1^ Institute of Translational Medicine, The Affiliated Hospital of Qingdao University, College of Medicine, Qingdao University, Qingdao, China; ^2^ Science and Technology Department, Qingdao University, Qingdao, China; ^3^ State Key Laboratory of Cardiovascular Disease, Heart Failure Center, Fuwai Hospital, National Center for Cardiovascular Diseases, Chinese Academy of Medical Sciences, Peking Union Medical College, Beijing, China; ^4^ Cardiology Department, Heart Center of Fujian Province, Union Hospital, Fujian Medical University, Fuzhou, China

**Keywords:** ferroptosis, metabolism, tumor treatment, cancers, drug resistance

## Abstract

Ferroptosis is a new form of programmed cell death (PCD) characterized by an excess iron accumulation and subsequent unbalanced redox states. Ferroptosis is different from the already reported PCD and has unique morphological features and biochemical processes. Ferroptosis was first elaborated by Brent R. Stockwell’s lab in 2012, in which small molecules erastin and RSL-3 induce PCD in Ras mutant cell lines. Ferroptosis involves various physiological processes and occurrence of disease and especially shows strong potential in cancer treatment. Development of small molecule compounds based on Stockwell’s research was found to kill cancer cells, and some FDA-approved drugs were discovered to result in ferroptosis of cancer cells. Radiotherapy and checkpoint therapy have been widely used as a treatment for many types of cancer. Recently, some papers have reported that chemotherapy, radiotherapy, and checkpoint therapy induce ferroptosis of cancer cells, which provides new strategies for cancer treatment. Nevertheless, the limitless proliferation of tumor cells and the lack of cell death mechanisms are important reasons for drug resistance for tumor therapy. Therefore, we reviewed the molecular mechanism of ferroptosis and sensitivity to ferroptosis of different cancer cells and tumor treatment strategy.

## 1 Introduction

Cell death is the ultimate destination of all cells. It has been thought that cell death is not controlled by genes for a long time. Programmed cell death (PCD) is induced by specific extracellular or intracellular signals and regulated by death-related genes (1). With the deepening of cell death research, the concept of PCD has been continuously enriched and is still being expanded. Apoptosis is the first discovered PCD, in which apoptosis effector caspase is involved in this pathway. In recent years, many forms of PCD have been discovered, including necroptosis, autophagy, pyroptosis, and ferroptosis. Ferroptosis, as a new form of PCD, has gradually come into view and has become a research hotspot in the field of cell death.

Ferroptosis is triggered by the accumulation of excess iron and subsequent reactive oxygen species (ROS) production and is morphologically, biochemically, and genetically distinct from apoptosis, necrosis, and autophagy. Glutathione peroxidase 4 (GPX4) is a protein resistant to ferroptosis, which catalyzes the reduction reaction of lipid peroxide (LOOH) and maintains the balance of redox reaction. The deficiency of GPX4 causes ferroptosis owing to the imbalance of redox states. Iron is an important microelement to support life. Nevertheless, excess iron generates abundant ROS by Fenton reaction, which breaks redox balance and induces ferroptosis. Ferroptosis is involved in multiple physiological and pathological processes, such as intestinal, renal, and cardiac ischemia and reperfusion (I/R), neurodegenerative diseases, hemochromatosis, and cancer (2–6).

Malignant tumors are commonly referred to as “cancers.” According to the statistics of the World Health Organization, about 9.6 million people died of cancer in 2018, and nearly one-sixth of all deaths were caused by cancer worldwide. Malignant tumor is the second leading cause of death in the world and one of the major diseases that seriously threaten human life and health (7). Small molecular compounds erastin and RSL-3 trigger ferroptosis in Ras mutant cancer cell lines and lead to the death of cancer cells (8, 9). These findings provide a new strategy for cancer treatment by pharmacological or genetic intervention related to ferroptosis, which is a significant work in scientific research as well as pharmaceutical research. This paper will mainly describe the mechanism of ferroptosis and the research progress in cancer treatment, especially chemotherapy, radiotherapy, and tumor immunotherapy, which provides new ideas for cancer drug resistance in several aspects.

## 2 Overview of Ferroptosis

In 1959, Harry Eagle strived to search for necessary nutrients of cell metabolism and found only deficiency of cysteine inhibiting the growth of human and mouse cells. A cell lacking cysteine presents representative characteristics different from deprivation of other amino acids (10). Glutathione (GSH) synthesis inhibition and cysteine deficiency are certified to be important causes of cell death in the next few years (11). It is not recognized as ferroptosis until recent studies found that both iron chelators and lipophilic antioxidants prevent the occurrence of this kind of cell death (12).

Stockwell’s laboratory screened compounds lethal for HRAS mutant cell lines in a small-molecule pool and, in 2003, identified a compound named “erastin” which is proved to trigger ferroptosis (13). The system Xc-, Na+-independent cystine/glutamate antiporter is a direct target of erastin. Cells treated with erastin inhibit the function of system Xc-, which attenuates cystine uptake and GSH synthesis and leads to ferroptosis. Ferroptosis was finally named in 2012 and described as a regulated cell death characterized by excessive lipid peroxidation and redox active iron (9). Another well-known ferroptosis inducer RSL-3, a GPX4 inhibitor, induces ferroptosis selectively in HRASv12-mutant but not wild-type BJeLR-derived cell lines (8).

The most obvious morphological features of ferroptosis are shrunken mitochondria, increased membrane density, cristae degeneration and breakdown, and outer mitochondrial membrane rupture.

The morphology of the nucleus remains unchanged and without chromatin condensation appearance. However, margination, condensation, and fragmentation of chromatin are the main features during apoptosis (14). Besides, ROS augments peroxidation of membrane lipid and then damages the barrier function of the cytomembrane, but apoptosis involves apoptotic body production and without inflammation emergence (1). Necroptosis relies on a cascade pathway composed of receptor-interacting protein 1/3 (RIPK1/RIPK3) and mixed lineage kinase domain-like pseudokinase (MLKL) activation with the characteristics of membrane rupture and Annexin V-positive membrane bubble production, which is different from ferroptosis (1). Autophagy is another form of PCD characterized by the *de novo* synthesis of organelles enclosed by a double membrane and fused with a lysosome, which leads to their digestion. The main morphology of autophagy is also not present in ferroptosis (15). In all, the morphology of ferroptosis is sensibly different from that of apoptosis, necroptosis, and autophagy. Therefore, we compared the four different patterns of cell death, as shown in **Table 1**.

**Table 1:** Comparison of different types of cell death.

Type of cell death	Morphological features	Regulation genes	Activator
Apoptosis	Chromatin condensation and nuclear fragment; membrane blebbing and maintain integrity; apoptotic bodies formation	Caspase family; Bax family; BH3 family; Cyt c; *p53* and Bcl-2 family.	TNF family; FasL family
Necroptosis	Cell membrane breakdown; moderate chromatin condensation; swelling of organelles and cytoplasm	RIPK1; RIPK3; MLKL; flotillin and syntenin-1	TNF-α plus pan-caspase inhibitor treatment; HSV-1 infection; influenza virus infection and MCMV infection
Autophagy	Formation of double-membraned autophagic vesicle	ATG family; Beclin1; mTOR	Nutritional deficiencies; oxidative stress; amino acid starvation
Ferroptosis	Rupture of mitochondrial outer membrane, smaller mitochondria with increased density; nucleus remain intact	GPX4; ACSL4; SLC7A11; TfR1; FTH1; FSP1	erastin; FAC; RSL3; sorafenib

## 3 Metabolism and Ferroptosis Execution

The molecular mechanism of ferroptosis is mainly dependent on the production and elimination of lipid peroxidation, two competing biochemical processes in the cell. Iron and polyunsaturated fatty acids (PUFAs) are used as materials in the lipid peroxidation process to promote ferroptosis. GSH as a substrate of GPX4 clears peroxide and regulates ferroptosis negatively. When cells are unable to remove the excess peroxide effectively through an antioxidative mechanism, the accumulation of peroxidative lipids induces ferroptosis. Many physiological processes including iron metabolism, amino acid metabolism, and lipid metabolism can regulate ferroptosis by influencing cellular redox states.

### 3.1 Iron Metabolism

Iron originated from food is absorbed by intestinal epithelial cells of duodenal mucosa, where iron ion reductase catalyzes ferric iron (Fe^3+^) and reduces to Fe^2+^, and Fe^2+^ is transported into cells by divalent metal transporter 1 (DMT1) (16, 17). Extracellular Fe^3+^ combined with transferrin is transported into the cell with the help of cell surface receptor transferrin receptor 1 (TfR1). Heat shock protein family B member 1 (HSPB1) suppresses the TfR1 recycling and iron uptake. The deficiency of transferrin and TfR1 in glutamate-free culture medium significantly inhibits ferroptosis. Fe^3+^ is transported into the endosome and reduced to Fe^2+^ by STEAP family member 3 (STEAP3) in the cell (18). The reduced Fe^2+^ is transported into the cytoplasm with the help of DMT1 and becomes the main component of the iron pool (19). Intracellular iron is exported into the extracellular matrix and oxidized Fe^2+^ to Fe^3+^ in the meantime by ferroportin (FPN) to maintain normal levels of iron.

**Figure 1:**
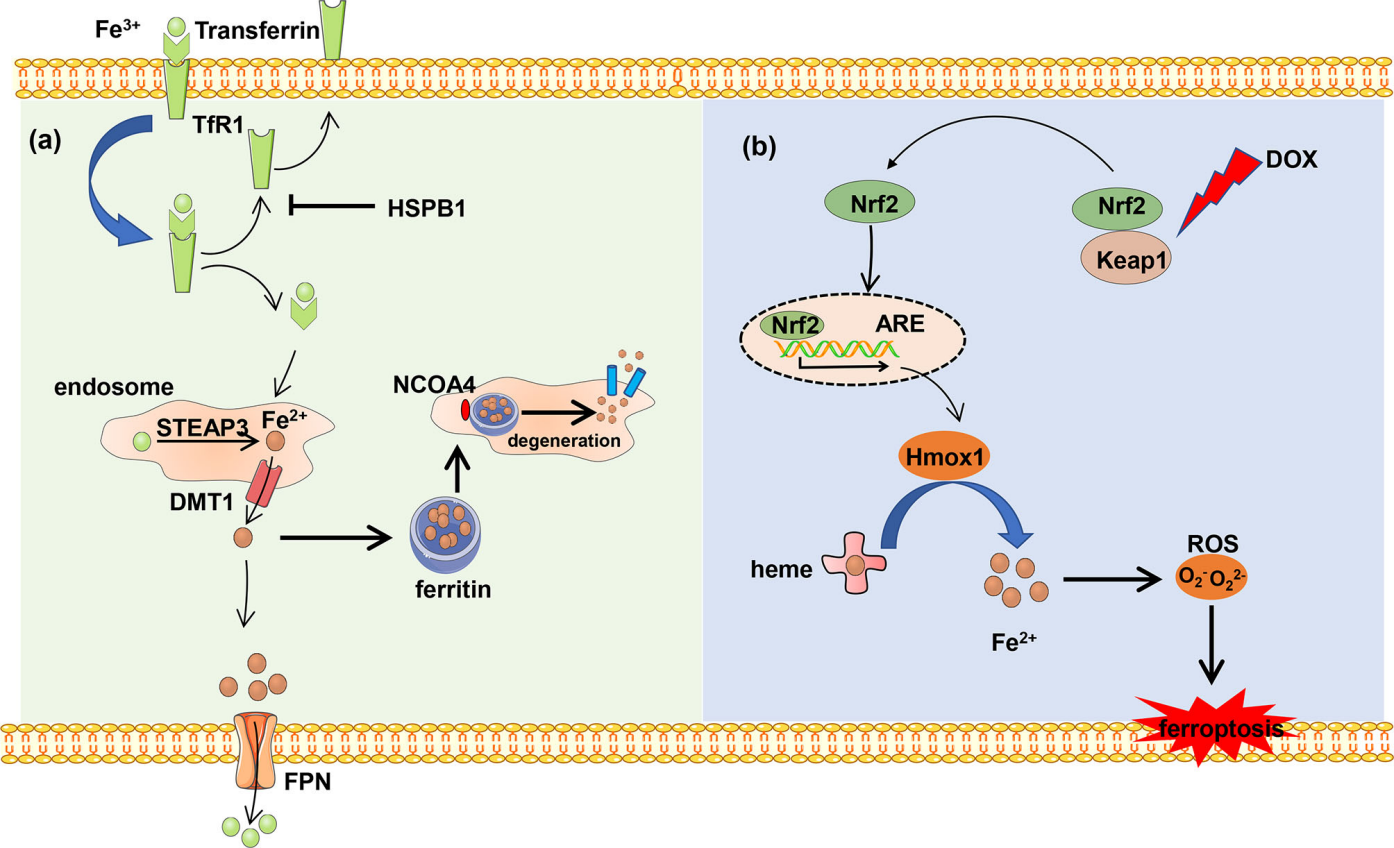
The metabolism of iron. **(A)** Ferric iron (Fe^3+^) coupled with transferrin and then was imported into intercellular circumstance by TfR1. Transferrin was recycled and exported out of the cell, which can be blocked by HSPB1. Fe^3+^ is reduced and forms reduced iron (Fe^2+^) by DMT1 in the endosome, and Fe^2+^ is transported into the cytoplasm. Excess Fe^2+^ is stocked in the ferritin to avoid iron overload. The stocked Fe^2+^ can be released from ferritin by NCOA4-mediated ferritinophagy. Besides, Fe^2+^ is exported out of the cell and is oxidized by FPN. **(B)** DOX induces ferroptosis. The heart-exported DOX activates the Keap1/Nrf2 pathway, and transcription factor Nrf2 activates downstream protein Hmox1 which oxidizes heme and release of iron which results in ferroptosis.

Excess iron generates ROS and then triggers ferroptosis. In order to avoid cell death, the excess iron is stocked in ferritin and produces redox-inactive ferritin heteropolymers to sustain the redox balance and protect against ferroptosis. Besides, the stocked iron is released from ferritin in which ferritin is degraded by nuclear receptor coactivator 4 (NCOA4)-mediated ferritinophagy to induce ferroptosis. NCOA4 interacts with HERC2 ubiquitin E3 ligase and is degraded by the ubiquitin proteasome degradation pathway to ensure a normal concentration. Under low cellular iron conditions, the interaction of NCOA4 and HERC2 is interrupted resulting in the elevation of NCOA4 expression, which increases ferritinophagy and iron levels (20). In all, NCOA4 overexpression increases the degradation of ferritin, which leads to upregulation of the iron concentration and promotes ferroptosis. On the other hand, the downregulation of NCOA4 inhibits degradation of ferritin and attenuates sensitivity of cells to oxidative damage (21) (**Figure 1A**).

Each heme consists of four pyrrole subunits forming a ring with a ferrous ion at the center. The heme oxygenase enzyme 1 (Hmox1) catalyzes the region-specific hydroxylation of heme and produces biliverdin, carbon monoxide, and Fe^2+^. In some pathological states such as doxorubicin (DOX)-induced cardiomyopathy and cardiac ischemia/reperfusion (I/R), Hmox1 is significantly upregulated. Non-heme iron is accumulated by degrading heme *via* the nuclear factor E2-related factor 2 (Nrf2)-mediated upregulation of Hmox1. The excess iron induces ferroptosis and cardiac injury which can be blocked by ferroptosis inhibitors ferrostatin-1 and iron chelation dexrazoxane (**Figure 1B**).

### 3.2 Amino Acid Metabolism

**Figure 2:**
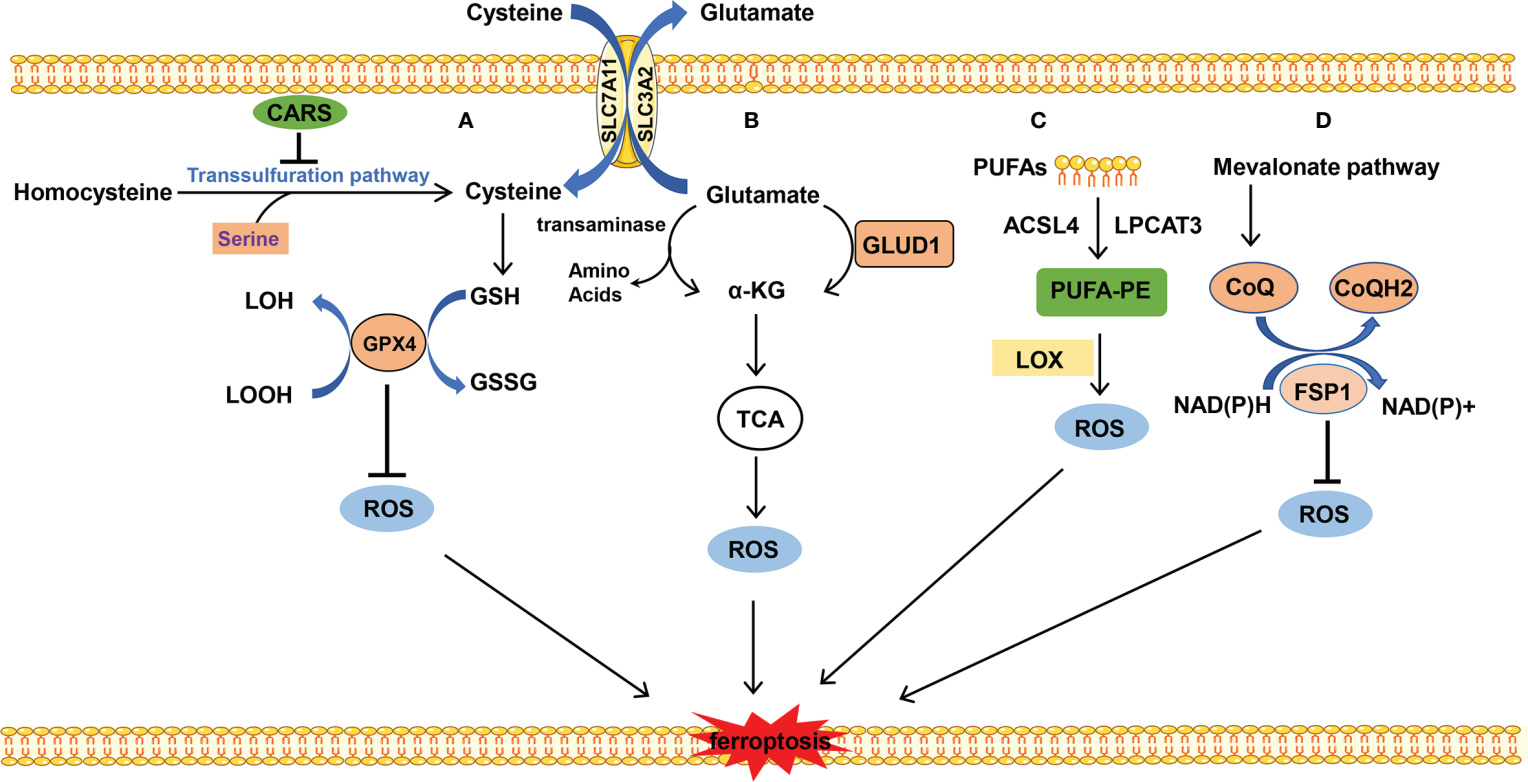
The metabolism of amino acids and lipid. **(A)** Cysteine is transported into the cell, and glutamate is transported out of the cell by system Xc- at the same time. Cysteine is used to synthesize GSH to maintain the balance of redox states. Besides, cysteine can be synthesized by the transsulfuration pathway, which is blocked by CARS. **(B)** Glutamate is converted into α-KG by transaminase or GLUD1 pathway and participates in TCA, which results in ROS production. **(C)** PUFAs which mainly stem from the cellular membrane are catalyzed into PUFA-PE by ACSL4 and LPCAT. PUFA-PE is peroxided by the LOX family. **(D)** FSP1 depends on the balance of redox. CoQ is synthesized by the mevalonate pathway and plays an important role in the CoQ antioxidant system.

GSH is a substrate of GPX4 which maintains the redox balance in cells and determines whether cells undergo ferroptosis or not. GPX4 uses 2-molecule GSH as an electron donor to reduce toxic lipid peroxides (L-OOH) to non-toxic fatty alcohols (L-OH), and GSH is oxidized into oxidized glutathione (GSSG) at the same time. GSSG can be reduced by NADPH to GSH and is then involved in redox reaction. GSH is a tripeptide containing the γ-amide bond and sulfhydryl group and composed of glutamic acid, cysteine, and glycine. Cysteine serves as the rate-limiting precursor for GSH. Excess methionine is converted into cysteine through transsulfuration in mammals to initiate cysteine formation. The cystathionine β-synthase enzyme catalyzes serine with the methionine cycle intermediate homocysteine to form cystathionine, which is subsequently cleaved by cystathionine γ-lyase to release cysteine (22). When cystine is deficient, some cells synthesize cysteine through methionine transsulfuration pathways bypassing the system Xc- and therefore resist to ferroptosis caused by system Xc- inhibition (23). It is reported that knockdown of cysteinyl-tRNA synthetase (CARS) leads to enhanced activity of transsulfuration and inhibits ferroptosis induced by erastin, in which the lipid ROS is prevented without altering iron homeostasis (24). However, most cancer cells need specific transporter-system Xc- (composed by SLC3A2 and SLC7A11 connected with disulfide bond) to import cystine which is converted to cysteine through an NADPH-consuming reduction reaction and is used for GSH synthesis (25). Inhibition of system Xc- leads to the imbalance of amino acid metabolism and subsequent ferroptosis. Cystine is directly transferred into the cell through the alanine, serine, cysteine-preferring system (ASC system) when the cell is in a reducing state. ASC system activation inhibits ferroptosis induced by erastin (18) (**Figure 2A**).

Glutamine is the most abundant amino acid which is used for biosynthesis of nucleotides, as a nitrogen source, and in the tricarboxylic acid cycle as a carbon source. The uptake of glutamate relies on the receptor complex of SLC38A1 and SLC1A5. The inhibition of SLC1A5 by chemical compound L-g-glutamyl-p-nitroanilide (GPNA) or RNA interference blocks ferroptosis, indicating that glutamine is indispensable for ferroptosis (26). The glutamate deamination mediated by glutamate dehydrogenase (GLUD1) or transamination pathway catalyzes glutamate to generate α-ketoglutarate (α-KG). Transaminase inhibitor amino-oxyacetate (AOA) or RNAi of the transaminase GOT1 inhibits ferroptosis. α-KG in combination with dialyzed FBS induces ferroptosis even during co-treatment with AOA in case of amino acid starvation. It is indicated that downstream metabolites of glutaminolysis α-KG play an important role in ferroptosis (27). Besides, high-concentration glutamate inhibits the function of system Xc- and results in ferroptosis, which may explain the neurotoxicity of glutamate when glutamate is accumulated at a high concentration (9) (**Figure 2B**).

### 3.3 Lipid Metabolism

ROS are a group of molecules with partially reduced oxygen, including peroxides, superoxides, singlet oxygen, and free radicals, which are detrimental to DNA or proteins. One of the sources of ROS production is lipid peroxidation. It is reported that lipid peroxides cause cell damage in many ways. Firstly, lipid peroxides are further decomposed into ROS, which further amplifies the lipid peroxidation process. Secondly, lipid peroxidation changes the physical structure of the membrane, such as the thickness and bending degree of the membrane, or by forming holes in the membrane, releasing harmful substances out of the cell. Thirdly, the by-products (aldehydes) of lipid peroxidation cause cell damage, such as malondialdehyde (MDA) and 4-HNE (28, 29). Therefore, the accumulation of lipid peroxides, especially phospholipid peroxides, is considered to be the hallmark event of ferroptosis (30).

The substrate of lipid peroxidation is fatty acid, and polyunsaturated fatty acids (PUFAs) are more prone to be oxidized compared with saturated fatty acids and monounsaturated fatty acids (30), in which the free PUFA is esterified into membrane phospholipids which are further oxidized to induce ferroptosis. This enzymatic reaction process of lipid peroxidation is mediated by long-chain lipidyl coenzyme A ligase 4 (ACSL4) (31), lysophosphatidylcholine acyltransferase 3 (LPCAT3) (32), lipoxygenase (LOX) (33), and so on. ACSL4 converts free long-chain fatty acids into fatty acyl-CoA esters and plays a key role in lipid biosynthesis and fatty acid degradation. LPCAT3 possesses transferring acyl groups and 1-acylglycerophosphocholine O-acyltransferase activity. ACSL4 and LPCAT3 are involved in the synthesis of PUFA-phosphatidylethanolamine (PUFA-PE) in cellular membranes. The former is involved in catalyzing the esterification reaction, especially preferring to esterify the acyl of arachidonic acid. The latter helps esterify the acyl of arachidonic acid to insert it into the membrane phospholipids (32). Subsequently, LOX mediates the peroxidation of membrane phospholipids. Cells with LOX overexpression tend to have stronger lipid peroxidation and are more sensitive to erastin- or RSL3-induced ferroptosis, although to a slightly different degree (34, 35). Besides, it is reported that GPX4 and ACSL4 double-knockout cells are resistant to ferroptosis (31). This process prefers to use free PUFA as substrate rather than phospholipids containing PUFA (36) (**Figure 2C**).

### 3.4 The Others

Besides iron, lipid, and amino acid metabolism, ferroptosis is also regulated by ferroptosis suppressor protein 1 (FSP1), Nrf2, heat shock protein (HSP), and so on. Particularly, FSP1 catalyzes the regeneration of CoQ10 using NAD(P)H and the reduction form of CoQ10 traps lipid peroxyl radicals that mediate lipid peroxidation, which inhibits ferroptosis (37). Nrf2 is an important transcription factor, which activates the antioxidant genes expression (38). HSPB1 regulates iron homeostasis and prevents ROS production caused by the high concentration of intracellular iron (39).

#### 3.4.1 FSP1-Involved Ferroptosis

FSP1 is a recently discovered important ferroptosis regulator. Previously, GPX4 was thought of as a central mechanism for ferroptosis inhibition. Some researchers have tried to find other protective mechanisms parallel to GPX4-inhibition-mediated ferroptosis, in other words, whether cells can survive in ferroptosis-inducing conditions and without GPX4 participation. Coincidentally, the two research groups discovered FSP1 almost simultaneously (37, 40). FSP1 is a negative regulator of ferroptosis parallel to GPX4. Mechanistically, the myristoylation of FSP1 mediates the recruitment of FSP1 to lipid droplets and plasma membrane where FSP1 mediates the NADH-dependent reduction of coenzyme Q (CoQ) which functions as a radical-trapping antioxidant and suppresses the generation of lipid peroxides. The mevalonic acid pathway plays an important role in the upstream of CoQ synthesis; small molecular compound FIN56 induces ferroptosis by mevalonic acid pathway inhibition. In addition, supplementation of cells with edibenzoquinone (an analogue of CoQ) protects against the lethality of FIN56 (41) (**Figure 2D**).

#### 3.4.2 Nrf2-Regulated Ferroptosis

Nrf2 is a transcription factor, closely related with ferroptosis by regulating the intracellular oxidation homeostasis and controlling lipid peroxidation. Nrf2 inhibits ferroptosis by regulating the expression of downstream proteins such as metallothionein 1G (MT-1G), SLC7A11, and Hmox1. It is reported that Nrf2 inhibits ferroptosis and protects against acute lung injury due *via* regulating SLC7A11 and Hmox1 (42). Besides, the Nrf2/Hmox1 pathway participates in heme degradation and iron release and causes ferroptosis of the heart in doxorubicin-induced cardiomyopathy (4). Activation of Nrf2 is essential for induction of MT-1G expression and ferroptosis inhibition following sorafenib treatment, which is an important mechanism of sorafenib resistance in hepatocellular carcinoma therapy (43).

#### 3.4.3 HSPB1-Relied Ferroptosis

HSP is expressed constitutively under normal conditions, but overexpression is induced under stress conditions. HSPB1 also called human HSP27 or mouse HSP25 is a member of HSPs and plays a negative role in ferroptosis of cancer cells. HSF1 is a master regulator of HSP expression (44). It is reported that protein kinase C-mediated phosphorylation of HSPB1 inhibits ferroptosis by reducing lipid ROS production. Erastin-induced ferroptosis is enhanced by knockdown of HSP1 and HSPB1. Consistent with this phenomenon, inhibition of HSF1-HSPB1 and phosphorylation of HSPB1 increase the anticancer activity of erastin *in vivo (* 45).

In all, the abnormal metabolism of iron, amino acid, and lipid which are important factors to trigger ferroptosis is shown. Some factors such as FSP1, Nrf2, and HSPB1 recently have proved to participate in ferroptosis.

## 4 Organelles Involved in Ferroptosis Regulation

Eukaryotic cells are organized by membrane-bound organelles which maintain intracellular homeostasis. Stress conditions lead to the dysfunction of cell organelles and augment of cell death. Some cell organelles were reported to involve in ferroptosis regulation such as mitochondria, endoplasmic reticulum (ER) and lysosome. Hereon, we will discuss the specific functions of different organelles in the regulation of ferroptosis.

### 4.1 Mitochondria

Mitochondria are also called the powerhouse, which provide energy for the cell and are a site of aerobic respiration. The researchers have found that mitochondria are involved in the regulation of apoptosis. However, the functions of mitochondria in ferroptosis remain unknown. Gao et al. have found that mitochondria play an important role in ferroptosis induced by cysteine deprivation and are dispensable in GPX4 inhibition-induced ferroptosis. It is likely that mitochondria function upstream of GPX4 and promote the exhaustion of GSH under cysteine deprivation conditions. The production of glutaminolysis α-KG replenishes the tricarboxylic acid (TCA) cycle intermediates which are involved in cysteine deprivation-induced ferroptosis. The TCA cycle and electron transport chain (ETC) are the main functions of mitochondria which play roles as a major resource for lipid ROS production in cysteine-deprivation induced ferroptosis (46). The function of mitochondria in ferroptosis regulation is confirmed in DOX-induced cardiomyocyte ferroptosis in which the lipid peroxidation and iron are increased in the mitochondria (4). Besides, mitochondrial ATP–binding cassette (ABC) transporters play an important role in regulating cellular iron metabolism and maintenance of redox status. Deficiency of mitochondrial ABC transporters impairs the activity of mitochondrial ETC, iron overload, and elevation of ROS which results in mitochondrial dysfunction (47).

### 4.2 Lysosome

Lysosome contains a variety of hydrolytic enzymes which hydrolyze a variety of exogenous and endogenous macromolecular substances to maintain intracellular homeostasis by multiple pathways especially autophagy (48). Lysosomal cell death is mainly executed by cathepsin and induced by iron overload or oxidative injury (49). It is reported that ferroptosis differs from lysosomal cell death and autophagy. However, elevation of autophagy and lysosomal activity is related with the induction of ferroptosis (50, 51). The NCOA4-mediated degradation of ferritin promotes ferroptosis, which is related with lysosome (52). STAT3 as an important transcription factor that regulates lysosomal cell death *in vitro* and *in vivo* by mediating the expression of cathepsin B, which is indispensable for ferroptosis (53).

### 4.3 Endoplasmic Reticulum

The alterations of redox state and calcium levels and failure of secretory proteins post-translational modification activate unfolded protein response (UPR) to defend against ER stress. Recent studies reveal that the ER stress response induced by the ferroptotic agent plays an important role in the cross talk between ferroptosis and apoptosis. Erastin induces ER stress and promotes the expression of the *p53*-upregulated modulator of apoptosis (PUMA) pathway *via* transcription factor C/EBP-homologous protein (CHOP) (54). p38 phosphorylates and activates CHOP, which causes an elevated expression of Bim and DR5 and induces apoptosis. PUMA is inactive after treatment with the ferroptic agent while PUMA transforms into an active state after combined treatment with both erastin and TRAIL. This conversion of PUMA from an inactive to active state determines whether necrosis or apoptosis occurs (55).

## 5 Ferroptosis in Cancer Treatment

The treatment of malignant tumors has been accelerating and bringing obvious clinical prospects for patients in recent years. The global expenditure on oncology drugs and the complexity of clinical trial activities are increasing dramatically. Despite the high level of basic and clinical research, tumor therapy remains one of the most challenging areas of research with a significant risk of failure and long development period. At present, surgery, chemotherapy, radiotherapy, and immunotherapy are the main strategies for the treatment of cancer (56). Even though a wide range of clinical antitumor therapies was developed, patients are still subjected to poor efficacy of treatment, recurrence, and metastasis, and eventually death due to treatment failure. How to overcome tumor therapeutic resistance and find new therapy strategy has become a clinical problem to be solved urgently. A recent study found that combined treatment with both ferroptosis inducer erastin and antitumor drugs enhances the antitumor activity of drugs, which provides great clinical value in the treatment of tumor (57–61). All the details are shown in **Table 2**.

**Table 2:** Drugs and compounds that inhibit tumor growth.

Drugs or compounds	Targets protein	Targeted tumors
Erastin	VDAC2/3	Ras mutated cell lines
RSL-3	GPX4	Renal cancer cell; human non-small cell lung cancer; glioma cell; acute lymphoblastic leukemia; rhabdomyosarcoma cells; melanoma; fibrosarcoma cell
Sorafenib	System Xc-	Renal cancer cells; human hepatocellular carcinoma; pancreatic cancer cells; human non-small cell lung cancer
Sulfasalazine	GPX4	Glioma cells; head and neck cancer; fibrosarcoma cell; diffuse large B-cell lymphomas; breast cancer
Artemisinin	GPX4	Lung cancer cell; human colorectal cancer cells; human breast cancer cell
BSO; DPI2; FIN56	GPX4	Fibrosarcoma cell
Paclitaxel	GLS, SLC7A11, SLC1A5, p53	Colorectal carcinoma cells
DHA	Ferritin	AML
Cisplatin	GPX4	Lung cancer cells

### 5.1 Chemotherapy

At present, many small-molecule compounds have been proved to induce the death of tumor cells by inducing ferroptosis. Therefore, ferroptosis inhibitors are expected to be developed into new antitumor small-molecule drugs. According to the different targets of compounds, the ferroptosis inducer can be divided into four categories: (1) system Xc- inhibitor; (2) GPX4 inhibitor; (3) GPX4 and CoQ degradation inducer; and (4) lipid peroxide inducer. It is worth mentioning that some FDA-approved clinical drugs are proved to induce ferroptosis, which extends its clinical application value.

Sorafenib is an oncogenic kinase inhibitor which has been approved as an anticancer drug in the clinical treatment. The cytotoxic effect of sorafenib on hepatocellular carcinoma (HCC) cells is restrained iron chelation deferoxamine. Importantly, sorafenib induction of ferroptosis in cancer cells is unrelated with inhibition of the RAF-MEK-ERK kinase cascade. Lachaier et al. show that sorafenib blocks the system Xc- activity and the synthesis of GSH in a similar way as erastin to induce ferroptosis in different solid tumors (62).

Sulfasalazine (SAS) is a sulfonamide-based antimicrobial agent which is used to treat chronic inflammation of the gut, joints, and retina. A recent study reveals that sulfasalazine inhibits breast cancer cell viability by ferroptosis with the characterization of an abnormal increase in ROS and inhibition of GPX4 and system Xc-. What is more, liproxstatin-1 inhibits the SAS-induced increase in ROS. Importantly, sulfasalazine is more likely to trigger ferroptosis in breast cancer cells, especially in cells with low estrogen receptor expression (63). Inhibition of system Xc- by SAS overcomes the cisplatin resistance of head and neck cancer (HNC) cells by inducing ferroptosis (57, 58).

Artemisinin is the most effective drug for treating malaria resistance. Nowadays, artemisinin and its derivatives have been explored as potential anticancer agents. Dihydroartemisinin (DAT) is a derivative of artemisinin which induces lysosomal degradation of ferritin and increases the cellular free iron level (64). The binding of iron regulatory proteins (IRPs) with mRNA molecules containing iron-responsive element (IRE) sequences is an important iron homeostasis mechanism. DAT impairs IRP/IRE-controlled iron homeostasis and further increases cellular free iron. Besides, DAT promotes ferroptosis of a cohort of cancer cells mediated by GPX4 inhibition, which are originally resistant to ferroptosis (65). Artesunate-induced ferroptosis in Burkitt lymphoma DAUDI and CA46 cells results in ER stress response and activation of the ATF4-CHOP-CHAC1 pathway, which is blocked by liproxstatin-1, ferrostatin-1, and deferoxamine (DFO) (66). A previous study has reported that the antitumor effect of artesunate relies on mRNA upregulation of iron-related genes and induces ferroptosis in an iron-dependent manner (67).

Paclitaxel (PTX) is a natural alkaloid isolated from the bark of *Taxus brevifolia*. Low-dose PTX is a promising treatment for some cancers, especially colorectal carcinoma cells. The expression of glutaminolysis-related genes GLS, SLC7A11, and SLC1A5 is reduced after low-dose PTX treatment in colorectal carcinoma cells, and the expression of tumor-suppressor genes p53 and p21 is elevated at the same time. Low-dose PTX increases lactate production and decreases the pH of tumor microenvironments, which results in the inhibition of tumor cell growth (68).

Dihydroartemisinin (DHA) is able to inhibit the growth of tumor cells including acute myeloid leukemia (AML). DHA arrests the tumor cell cycle at the G0/G1 phase and inhibits AML cell viability. Besides, DHA induces ferroptosis in an iron-dependent manner by regulating the activity of the AMPK/mTOR/p70S6k signaling pathway and promoting the autophagy degradation of ferritin and leads to an increase in the iron pool and elevates the accumulation of ROS (69).

Cisplatin is a first-line therapy for tumor. Cisplatin was found as an inducer of both ferroptosis and apoptosis among the five chemotherapeutic drugs in A549 and HCT116 cell lines. Cancer cells treated with cisplatin show downregulation of GSH and inactivation of GPX4, which results in initiation of ferroptosis. Combination therapy of cisplatin and erastin significantly improves antitumor efficiency (70). PRLX93936 is an analogue of erastin, which has been tested in clinical trials. Co-treatment of cisplatin and PRLX93936 induces the production of lipid peroxidation and Fe2+ and eventually promotes ferroptosis (71).

Besides the above drugs already used in the clinic, many small molecular ferroptosis compounds still stay in a laboratory research phase. Erastin was first found to induce ferroptosis. The chemosynthesis of more soluble erastin derivatives has been shown to have a more effective treatment effect in animal disease models of fibrosarcoma and diffuse large B-cell lymphoma (9). RSL-3 is a representative of the second class of compounds, which can induce ferroptosis in fibrosarcoma. Mechanically, RSL-3 targets the nucleophilic active site of GPX4 to inhibit the activity of GPX4 and induce ferroptosis (8). FIN56 triggers ferroptosis through a mechanism involving the regulation of GPX4 degradation. FIN56 also binds to and activates squalene synthase, an enzyme involved in isoprenoid biosynthesis, which leads to the lack of CoA and promotes ferroptosis (41). 1,2-Dioxolane is an organic peroxide and induces ferroptosis by influencing iron levels and inactivating GPX4, which is the fourth class of small-molecule drug (72).

### 5.2 Tumor Multidrug Resistance

Chemotherapy is one of the main therapeutic strategies for malignant tumors, but the phenomenon of multidrug resistance (MDR) has become the main reason for the failure of chemotherapy in tumor patients (73). At present, it is widely believed that MDR in tumors can be divided into two cases. The first case is known as intrinsic resistance, in which the tumor cells and tissues are resistant to the drug before receiving chemotherapy (74). The other is known as acquired resistance, that is, drug resistance in patients after effective chemotherapy (75). In recent years, more and more studies have been focused on how to effectively overcome MDR, and with ferroptosis coming into our sight, we will see the light for overcoming MDR.

Recent studies have demonstrated that restraint of ferroptosis is important for drug resistance. Treatment of lung or breast cancer cells with PTX or DOX activates NRF2 and leads to MDR. The abnormal activation of NRF2 caused by Keap1 mutation and p62 upregulation protects cancer cells against ferroptosis. The activation of NRF2 leads to an increase in downstream protein Fth1 and SLC7A11 upregulation, which causes ferroptosis resistance (76). Generally, cancer cells with high levels of GSH and SLC7A11 show higher MDR (56). Therefore, exploring small-molecule inhibitors targeting Keap1/NRF2 against MDR is of therapeutic significance. Pathways to increase intracellular iron concentration such as elevation of iron uptake by TfR1 overexpression, reduction of iron storage by ferritin knockdown or induction of ferritinophagy, and decrease of iron export enhance ferroptosis sensitivity and inhibit MDR, but on the other, reduction of iron results in ferroptosis resistance (77).

The current strategies of MDR include inhibition of the ABC transporter, development of new drug dosage forms, targeting of the tumor microenvironment, and regulation of ROS. Especially, ROS produced during ferroptosis may be destined to have border applications. A large number of studies have demonstrated that compounds regulating cellular ROS levels can enhance the death of MDR cancer cells and make MDR cancer cells sensitive to certain chemotherapy drugs (78). Besides, some redox-regulating enzymes, including mitochondrial ETC complexes, NADPH oxidases (NOXs), system Xc-, thioredoxin reductases, and Nrf2 play important roles in regulating cellular ROS levels and drug resistance as well as their clinical significance (79). Zhang et al. found that cisplatin significantly increased the expression of system Xc- and subsequently increased in GSH levels in tongue squamous cell carcinoma (TSCC). The upregulation of system Xc- and intercellular GSH levels contributed to cisplatin resistance in TSCC cells. Consistently, system Xc- suppression sensitizes TSCC to cisplatin treatment (80). The GPX4 inhibitor RSL-3 enhances the antitumor efficacy of cisplatin by increasing the accumulation of ROS and augmenting the level of iron in tumor cells to activate ferroptosis (81). Besides, activation of the Nrf2-ARE pathway resists GPX4 inhibition of HNC, and inhibition of Nrf2-ARE pathway sensitizes to ferroptosis in HNC (82).

The development of ferroptosis inhibitors and inducers accelerates the research progress in the mechanism study of ferroptosis and chemosensitivity. Ferroptosis inducer sulfasalazine enhances the antitumor sensitivity of cisplatin in HNC and rectal cancer (57). It is reported that different doses of ferroptosis inducer erastin are slightly different. For example, erastin can induce ferroptosis in the AML cell line at the concentration of 5 μmol/l and enhance the antitumor efficiency of cytosine arabinoside and doxorubicin at 1.5 μmol/l (83). Hangauer et al. reported that drug resistance cell lines are vulnerable to GPX4 inhibition. Inhibition of ATF4/HSPA5/GPX4 and system Xc- elevates the sensitivity of pancreatic ductal carcinoma to cisplatin and gemcitabine (84). The depletion of GSH caused by cisplatin and inactivation of GPX4 plays an important role in cisplatin-induced PCD of the non-small cell lung cancer cell line (70). Inhibition of the STAT3/Nrf2/GPX4 pathway promotes the sensitivity of cisplatin-resistant osteosarcoma to cisplatin (85).

### 5.3 Radiotherapy

Radiotherapy (RT) is one of the most effective cancer treatment methods; RT is the first-line treatment strategy for about 60% of cancer patients. The main mechanism of RT induced is summarized as follows. Firstly, RT induces oxidative damage by radiolysis of cellular water and stimulation of oxidase in all cellular compartments, including the lipid membrane. The accumulation of lipid peroxidation has been implicated to cause ferroptosis, a new form of PCD (9). However, the hypoxic environment in solid tumors limits the effectiveness of RT, because even though the double-stranded structure of DNA is damaged when exposed to RT, hypoxic conditions repair the ROS-induced double-stranded breaks in DNA. Therefore, the O2 concentration of the tumor microenvironment is elevated *in vivo*, so as to change the hypoxia state of tumor cells and promote RT sensitization (86). In addition, tumor cells with RT will release microparticles into the extracellular environment, which can enhance the effect of radiotherapy by inducing ferroptosis (87). What is more, RT induces intracellular mitochondria DNA damage, thereby activating the STING1/TMEM173-mediated DNA sensing pathway, leading to autophagy-dependent ferroptosis *via* lipid peroxidation (88).

Ivanov et al. found that treatment with iron-containing water for a long period before RT accelerated glioma death in rats and elevated the efficiency of radiotherapy due to the combination of apoptosis and ferroptosis, which can be blocked by iron chelator DFO (89). Lei et al. found that IR or deficiency of Keap1 promotes the expression of SLC7A11, which enhances radioresistance by inhibiting ferroptosis. Ferroptosis inducers such as RSL3, erastin, sorafenib, and sulfasalazine or inactivating SLC7A11 and GPX4 sensitizes radioresistant cancer cells (90). Besides, it has been reported that gold nanoparticles (AuNPs) induce the production of ROS under X-ray and UV radiation, which indicated that AuNPs can be used as radiosensitizer potential (90, 91). RGD/P-AuNPs formed by the combination of arginine-glycine-aspartic acid (RGD) tripeptide and polyethylene glycolated gold nanoparticles (P-AuNPs) inhibit the invasion of breast cancer cells after radiotherapy (92). Combination therapy of internalized RGD and RGD-conjugated mesoporous silica-coated gold nanorods with radiotherapy can cooperatively induce the G2/M phase arrest of MDA-MB-231 cells and promote ROS production, which enhances the radiosensitivity of breast cancer cells (93).

### 5.4 Immunotherapy

The tumor microenvironment includes tumor cells, tumor vascular system, extracellular matrix, and immune cells which are important factors in the therapeutic effect of tumor. Hypoxia, chronic inflammation, and immunosuppression are the main characteristics of the tumor environment. The expression of programmed death-ligand 1 (PD-L1) is upregulated in tumor cells to escape immunological surveillance, which interacts with PD-1 of the T cell surface receptor to trigger immune checkpoint response (94). Therefore, anti-PD-1 and anti-PD-L1 immunotherapy has been the most remarkable promise in treating tumors. However, immunotherapy cannot obtain the expected antitumor effect because the physical barrier composed of the fibroblast and dense extracellular matrix in the tumor microenvironment blocks the drugs delivery (95).

Immunotherapy-activated CD8^+^ T cells enhance specific lipid peroxidation related with ferroptosis in tumor cells, which benefits with the antitumor effect of immunotherapy. Mechanistically, CD8^+^ T cell downregulates the expression of system Xc- subunits SLC7A11 and SLC3A2 by releasing interferon γ (IFN-γ), which reduces cystine uptake and promotes lipid peroxidation in tumor cells. Depletion of cystine combines with PD-L1 blockade therapy and synergistically induces ferroptosis, which elevates T cell-mediated antitumor immunity (96). In conclusion, ferroptosis inducer and immune checkpoint inhibitor combination therapy will be very promising in the future. Damage-associated molecular pattern molecules (DAMPs) released from irradiated tumor cells and combined therapy of immune checkpoint with the TGF-β inhibitor transform procarcinogenic M2 macrophage into antitumor M1-polarized macrophage (87). A recent study has found a biomimetic magnetosome composed of a Fe3O4 magnetic nanocluster as core and pre-engineered leukocyte membranes as the cloak. The TGF-β inhibitor is anchored inside the membrane, and the PD-1 antibody is loaded on the membrane surface. The biomimetic magnetosome will enter into the tumor where the PD-1 antibody and TGF-β inhibitor create an immunogenic microenvironment synergistically, which increases the content of H_2_O_2_ and promotes the Fenton reaction with irons released from the magnetic nanocluster (97). Sun et al. developed a sorafenib and chlorin e6-co-loaded ROS-responsive nanoparticle (NP-sfb/ce6) which strengthens the antitumor effect. With the irradiation of 660 nm, ROS is generated from chlorin e6 (ce6) and destroys the nanoparticles and, as a consequence, promotes sorafenib release from NP-sfb/ce6. The released sorafenib with lose-dose irradiation attenuates tumor progression by inducing powerful T cell antitumor immunity in local and systemic response, remodeling the tumor microenvironment and disrupting the interaction between CD8^+^ T cell and diverse immunosuppressive cells (98). These findings illustrate the relationship between ferroptosis and immunotherapy and lay a theoretical foundation of ferroptosis–immunotherapy synergism for the tumor treatment. It meanwhile provides a direction for further exploring the molecular mechanism of ferroptosis to promote the efficacy of immunotherapy.

### 5.5 The Detection of Ferroptosis

Ferroptosis is a kind of iron-dependent cell death, so the detection of iron content in tumor cells is an important way to detect ferroptosis. Phen Green SK probes are green permeability dyes used to detect intracellular iron in living cells by flow cytometry or confocal microscopy. Iron reacted with ferrozine to form a violet-colored complex and was determined by spectrophotometry at 560 nm. Serum non-heme iron was measured using iron/TIBC reagent designed by Pointe Scientific, Inc. (Canton, MI, USA), and was determined by the chromogen method (99). Lipid peroxidation is one of the features of ferroptosis, which can be detected by BODIPY 581/591 C-11 or DCFH-DA. BODIPY 581/591 C-11 is a lipophilic dye that accumulates in cell membranes. Light emission at 530 and 590 nm (red fluorescence) will be detected with light excitation at 488 and 568 nm. When the dye is oxidized, the emission light will migrate from 590 to 510 nm (green fluorescence), which can be detected by flow cytometry. DCFH-DA is hydrolyzed by intracellular esterase to produce DCFH which can be oxidized by ROS into green fluorescent DCF and monitored by fluorescence microscopy or flow cytometry (9). 4-Hydroxynonenal and malondialdehyde are endogenous products derived from the peroxidation of ω-6 PUFAs, which can be used as biomarkers of ferroptosis. The thiobarbituric acid colorimetric method and enzyme-linked immunosorbent assay are used to detect the content of MDA and 4-hydroxynonenal in the cells, tissues, and serum (4). Besides, the expression of some gene changes after ferroptosis. For example, the expression of ACSL4, PTGS2, and TfR1 upregulates but that of GPX4 and FtH1 downregulates in ferroptic cells (100). Although these genes are well known to be closely associated with ferroptosis, further research is needed to discover more specific biomarkers and testing tools that will be helpful in providing meaningful iron guidance to clinicians.

### 5.6 The Current Challenges of Ferroptosis

Ferroptosis is initially discovered as a form of PCD in 2012 and plays important roles in cancer treatment. Based on currently acknowledged pathways such as system Xc-, GPX4, FSP1, and iron metabolism pathway, a number of ferroptosis inducers are used in the fundamental research of cancer treatment. Ferroptosis inducers are better to combine with conventional therapy compared with sole application of ferroptosis inducers in tumor treatment, which significantly enhances therapeutic effects.

Although ferroptosis-based cancer therapy has made great progress, it still faces several challenges, especially with regard to regulatory mechanisms and potential applications in clinical oncology. The exact mechanism of ferroptosis is unclear and remains to be investigated, for example, whether ferroptosis is triggered by lipid peroxidation or the by-products of lipid peroxidation. What is more, ferroptosis is involved in the occurrence and development of many diseases, but whether ferroptosis is a pathogenic factor of these diseases remains to be studied. In addition, little study is focused on the physiological function of ferroptosis during growth and development. Studying the role of ferroptosis in growth and development will be a benefit for cancer treatment.

In addition, there are still unaddressed questions regarding the potential application of ferroptosis in cancer treatment, especially in the area of translational drugs. First is whether the ferroptosis inhibitors or inducers can be applied *in vivo* safely. Fortunately, sorafenib, sulfasalazine, and artemisinin mentioned in previous sections are currently being used in the clinical treatment of different diseases, but their application in clinical practice of tumor treatment requires further investigation. The sensitivity of different tumor cells to ferroptosis varies because of the expression levels of genes associated with ferroptosis. Therefore, it is important for patients to consider the type of tumor to be treated with ferroptosis-related drugs. Given that pharmacokinetic and physicochemical properties are important for the druggability of compounds, the current laboratorial ferroptosis inducers lack these first-class properties, which restricts its clinical application. Although the combination of ferroptosis with other treatments has been shown to improve the clinical outcomes of cancers, most of this is animal experiment and an optimal combination strategy involving ferroptosis and other treatments has not yet been identified. Although the combination therapy of ferroptosis with other treatments improves the clinical outcomes of cancers, most of this is animal experiment and an optimal combination strategy involving ferroptosis and other treatments needs further exploration.

### 5.7 Conclusions and Perspectives

Ferroptosis is a newly discovered form of PCD, and the study of its mechanism and application in diseases is developing rapidly. From the perspective of mechanism, the core of ferroptosis is the excessiveness of iron and the generation of lipid peroxides. Previous studies considered GPX4 as the core molecule of ferroptosis, but it was recently found that FSP1 was also involved in ferroptosis as a negative regulatory factor paralleled to GPX4, which greatly enriched the comprehension of ferroptosis. It may extend more ferroptosis regulators according to FSP1 regulation (101).

Although great progress has been made in tumor biology and therapeutics, there is still a long way to go before the battle against cancer is won. It is worth mentioning that not all cancer cells are sensitive to ferroptosis. Therefore, study of the ferroptosis sensitivity to cancer cells originating from different tissues will benefit clinical practice to a large extent. Ferroptosis is expected to be a new therapeutic target and has attracted more and more attention in the field of tumor biology and tumor therapy. Ferroptosis is a double-edged sword, and thus the potential cytotoxic effect of key proteins and pathways related with the ferroptosis inducer or inhibitor should be studied to ensure the tumor to trigger Fenton reaction. Further research into the mechanism of ferroptosis provides more potential therapeutic targets for clinical applications, which may lead to the development of new drugs and new therapeutic approaches. The dependence of tumor cells on iron makes them sensitize on iron overload and ROS accumulation. Targeting ferroptosis as new antitumor treatment strategy has important significance. What is more, a cross-disciplinary combination of technologies leads to better antitumor therapies. For example, ferroptosis-related nanometer materials or ferroptosis inducer and chemotherapy or radiotherapy or immune checkpoint combination therapy is used for tumor treatment. Above all, as the study of iron death deepened, the drugs and nanomaterials targeting ferroptosis of tumor cells will have a good prospect, which will become an important field in cancer treatment.

## Author Contributions

KW put forward the theme of ferroptosis and cancers. JJ and HW developed the scope of the review. SW, TW, XY, FW, and XC carried out the PubMed searches. JJ and HW summarized the relevant papers, accomplished the figures, and wrote the manuscript. KW and CL compiled and approved the final version of the manuscript. Z-YC prepares and presents the published work by those from the original review article. Y-HZ takes the responsibility of preparation, creation or presentation of the published work, especially data presentation. All authors contributed to the article and approved the submitted version.

## Funding

This work was supported by the National Natural Science Foundation of China (81770275, 81770406, 81873472, 82070313) and Taishan Scholar Program of Shandong Province and Qingdao Public Domain Science and Technology Support Plan Project (19-6-1-6-nsh).

## Conflict of Interest

The authors declare that the research was conducted in the absence of any commercial or financial relationships that could be construed as a potential conflict of interest.

## Publisher’s Note

All claims expressed in this article are solely those of the authors and do not necessarily represent those of their affiliated organizations, or those of the publisher, the editors and the reviewers. Any product that may be evaluated in this article, or claim that may be made by its manufacturer, is not guaranteed or endorsed by the publisher.
